# PD-1/PD-L1 interaction up-regulates MDR1/P-gp expression in breast cancer cells via PI3K/AKT and MAPK/ERK pathways

**DOI:** 10.18632/oncotarget.21914

**Published:** 2017-10-20

**Authors:** Shengwei Liu, Shuang Chen, Weiguang Yuan, Hongyan Wang, Kewang Chen, Dianjun Li, Dalin Li

**Affiliations:** ^1^ Department of Immunology, Harbin Medical University and Heilongjiang Provincial Key Laboratory for Infection and Immunity, Harbin Medical University and Heilongjiang Academy of Medical Science, 150081, Harbin, China; ^2^ Department of Cancer Immunology, Cancer Institute of Harbin Medical University, Department of Cancer Immunology, Heilongjiang Academy of Medical Sciences, 150081, Harbin, China; ^3^ Institute of Harbin Hematology and Oncology, Harbin First Hospital, 150010, Harbin, China; ^4^ Department of Breast Surgery, Harbin Medical University Cancer Hospital, 150081, Harbin, China

**Keywords:** PD-1/PD-L1, MDR1/P-gp, PI3K/AKT signaling, MAPK/ERK signaling, breast cancer

## Abstract

Programmed cell death ligand 1 (PD-L1) is an immunosuppressive molecule expressed on tumor cells. By interacting with programmed cell death-1 (PD-1) on T cells, it inhibits immune responses. Because PD-L1 expression on cancer cells increases their chemoresistance, we investigated the correlation between PD-L1 and multidrug resistance 1/ P-glycoprotein (MDR1/P-gp) expression in breast cancer cells. Analysis of breast cancer tissues using tissue microarrays revealed a significant correlation between PD-L1 and MDR1/P-gp protein levels. Increased expression of PD-L1 was associated with lymph node metastasis and histological tumor grade. In addition, interaction of PD-L1 with PD-1 induced phosphorylation of AKT and ERK, resulting in the activation of PI3K/AKT and MAPK/ERK pathways and increased MDR1/P-gp expression in breast cancer cells. The PD-1/PD-L1 interaction also increased survival of breast cancer cells incubated with doxorubicin. These findings suggest that the PD-1/PD-L1 inhibition may increase chemotherapy efficacy by inhibiting the MDR1/P-gp expression in breast cancer cells.

## INTRODUCTION

Programmed cell death ligand 1(PD-L1), also known as B7 homologue 1 (B7-H1) or CD274, is an immune checkpoint molecule belonging to the B7 family [1]. PD-L1 is expressed on B cells, macrophages, dendritic cells (DCs), and T cells, as well as some non-hematopoietic cells [2]. Recently, PD-L1 has been found to be overexpressed on many different tumor cells, including bladder, ovarian, pancreatic, and breast cancer cells [3–6]. PD-1, also known as CD279, is the receptor of PD-L1; it is expressed by activated NK cells, dendritic cells, as well as T cells and B cells [7]. The binding of PD-L1 to PD-1 results in tyrosine phosphorylation of the PD-1 cytoplasmic domain and the recruitment of SH2-domain containing tyrosine phosphatase 2 (SHP2) [8]. This recruitment results in dephosphorylation of zeta-chain-associated protein kinase 70 (ZAP70), protein kinase C-θ (PKCθ), and CD3ζ, leading to attenuation of the T cell receptor/CD28 (TCR/CD28) signal [9], and inhibition of immune responses [10].

Breast cancer is diagnosed in nearly 1.4 million women every year and is a major cause of cancer-related death in women. The expression of PD-L1 is more frequent in basal breast cancers and basal type breast cancer cell lines [11, 12]. High expression of PD-L1 is associated with poor-prognosis characterized by progesterone receptor (PR)-negative, estrogen receptor (ER)-negative, and human epidermal growth factor receptor 2 (HER2)-positive status, large tumor size, and high proliferation and grade [13–15]. Compared to PD-L1 negative tumors, PD-L1 positive tumors exhibit an increased number of intra-tumor CD8 + T cells, indicating a strong association between the expression of PD-L1 and tumor-infiltrating lymphocytes (TILs) [16]. As the binding of PD-L1 to PD-1 results in dysfunction of cytotoxic T lymphocytes (CTL) and loss of antitumor immunity, reactivation of dormant TILs by PD-1/PD-L1 inhibitors could represent a promising strategy in PD-L1 up-regulated breast cancer.

Interestingly, recent reports indicate that PD-L1 also acts as an anti-apoptotic molecule on cancer cells. Knockdown of PD-L1 leads to an increase in spontaneous apoptosis and doxorubicin-induced apoptosis in breast cancer cells [17]. PD-L1 expression on mouse mast cells results in the resistance to CTL-mediated lysis and factor associated suicide ligend (FasL)-mediated apoptosis; the resistance requires intracellular domain of PD-L1 [18]. Binding of PD-L1 to recombinant PD-1 increases resistance to conventional chemotherapeutic agents in breast and prostate cancer cells, and ERK and mTOR phosphorylation [19]. It has been reported that interaction of PD-L1 and PD-1 activates PI3K/AKT pathway in myeloma and diffuse large B-cell lymphoma (DLBCL) [1, 20], but the mechanisms are poorly understood.

Overexpression of P-glycoprotein (P-gp), coded by *MDR1* gene, represents one of the mechanisms of how cancer cells reduce the intracellular concentration of anticancer drugs. P-gp is a 170 kDa plasma membrane glycoprotein that is a member of the ATP-binding cassette (ABC) transporter protein superfamily, and functions as an energy-dependent ATP efflux pump [21]. P-gp is widely distributed in normal tissues, including kidney, small intestine, liver, and brain, and likely protects these susceptible organs from toxic compounds [22]. P-gp is also highly expressed in multidrug–resistant cancer cells, and has an impact on the pharmacokinetics of a wide range of drugs, such as doxorubicin, epirubicin, etoposide, paclitaxel, and docetaxel. As PD-L1 expression on cancer cells increases their chemoresistance, we speculated that PD-L1 expression correlates with MDR1*/*P-gp expression in breast cancer cells.

In this study, we demonstrate that the PD-L1 expression correlates with the MDR1/P-gp expression in breast cancer tissues. In the presence of PD-1, PD-L1 up-regulates the MDR1*/*P-gp expression in breast cancer cells, and this up-regulation is mediated by the activation of PI3K/AKT and MAPK/ERK pathways.

## RESULTS

### Correlation of PD-L1 expression with MDR1/P-gp and clinicopathological features in breast cancer

Since MDR1/P-gp and PD-L1 play an important role in drug resistance, we investigated whether PD-L1 expression correlates with MDR1/P-gp in 150 breast cancer tissues. PD-L1 and MRD/P-gp protein expression was analyzed by immunohistochemistry (IHC) staining of tissue microarrays (Supplementary Figure 1). The levels of PD-L1 and MDR1/P-gp were defined as ‘low expression’ and ‘high expression’ according to the staining scores. High expression of PD-L1 was found in 105 breast cancer cases (70.0%). High expression of MDR1/P-gp was found in 74 breast cancer cases (49.3%). Low levels of PD-L1 and MDR1/P-gp were observed in 41 patients (27.3%), and high levels of PD-L1 and MDR1/P-gp were observed in 70 patients (46.7 %). Four patients (2.7%) had a low expression of PD-L1 and a high expression of MDR1/P-gp. Thirty-five patients (23.3%) had a high expression of PD-L1 and a low expression of MDR1/P-gp. Together, these results show that the expression of PD-L1 positively correlates with the expression of MDR1/P-gp in breast cancer tissues (R = 0.703, *p* < 0.01; Table 1).

**Table 1:** Correlation of PD-L1 and MDR1/P-gp expression

		PD-L1		Spearman R	*P* value
		low	high		
MDR1/P-gp	low	41	35	0.703	< 0.01^*^
	high	4	70		

^*^p < 0.01.

Next, we evaluated the correlation between PD-L1 expression and clinicopathological features in 247 breast cancer tissues. The protein levels of PD-L1 correlated with lymph node metastasis (*p* = 0.0009), and histological grade of tumors; PD-L1 was increased in grade I/II compared with grade III (*p* = 0.0022). In addition, Her-2 positive patients exhibited increased PD-L1 levels (*p* = 0.0043). No correlation was found with patients’ age, tumor node metastases (TNM) classification, and ER or PR status. We also investigated the correlation between PD-L1 expression and clinicopathological features in 59 triple-negative breast cancer (TNBC) patients. Increased expression of PD-L1 was associated with lymph node metastasis (*p* = 0.0362), suggesting that high levels of PD-L1 may promote lymph node metastasis in TNBC patients. Together, these data show that the PD-L1 expression is associated with lymph node metastasis, histological grade, and Her-2 status (Tables 2 and 3), suggesting that it may serve as a prognosis factor in breast cancer patients.

**Table 2:** Association of PD-L1 and clinicopathological features of breast cancer

ALL	Total no.	low (score < = 4)		high (score > 4)		*P* value
		*n*	%	*n*	%	
**Age**						
<= 50	143	57	23.08%	86	34.82%	0.9447
> 50	104	41	16.60%	63	25.51%	
**TNM stage**						0.5995
I	9	5	2.02%	4	1.62%	
II	162	64	25.91%	98	39.68%	
III	76	29	11.74%	47	19.03%	
**Lymph node metastasis**						0.0009^**^
Negative	150	72	29.15%	78	31.58%	
Positive	97	26	10.53%	71	28.74%	
**Grade**						
I	47	8	3.24%	39	15.79%	0.0022^*^
II	88	27	10.93%	61	24.70%	(I + II vs III)
III	29	21	8.50%	8	3.24%	
unknown	83					
**ER status**						
ER+	12	5	5.15%	7	7.22%	0.3349
ER-	85	48	49.48%	37	38.14%	
**PR status**						
PR+	8	2	2.06%	6	6.19%	0.0788
PR-	89	51	52.58%	38	39.18%	
**HER2 status**						
HER2+	26	8	8.25%	18	18.56%	0.0043^**^
HER2−	71	45	46.39%	26	26.80%	

^*^p < 0.05; ^**^p < 0.01.

**Table 3:** Association of PD-L1 and clinicopathological features of Triple-negative breast cancer (TNBC)

TNBC	Total no.	low (score < = 4)		high (score > 4)		*P* value
		*n*	%	*n*	%	
**Age**						
< = 50	36	25	42.37%	11	18.64%	0.4974
> 50	23	14	23.73%	9	15.25%	
**TNM stage**						
I	0	0	0.00%	0	0.00%	0.5110
II	33	23	38.98%	10	16.95%	
III	26	16	27.12%	10	16.95%	
**Lymph node metastasis**						0.0362^*^
Negative	40	30	50.85%	10	16.95%	
Positive	19	9	15.25%	10	16.95%	
**Grade**						
I	2	2	3.39%	0	0.00%	0.5096
II	27	15	25.42%	12	20.34%	(I + II vs III)
III	23	18	30.51%	5	8.47%	
unknown	7					

^*^p < 0.05; ^**^p < 0.01.

We then investigated the correlation between MDR1/P-gp expression and clinicopathological features in 160 breast cancer tissues. However, we found no significant correlation between MDR/P-g expression and clinicopathological features (Table 4).

**Table 4:** Association of MDR1/P-gp and clinicopathological features in breast cancer

		low (score < = 4)		high (score < = 4)		*P* value
ALL	Total no.	*n*	%	*n*	%	
**Age**						
< = 50	93	48	51.61%	45	48.39%	0.768
> 50	67	33	49.25%	34	50.75%	
**TNM stage**						
I	10	7	70.00%	3	30.00%	0.354
II	119	57	47.90%	62	52.10%	
III	31	17	54.84%	14	45.16%	
**Tumor size**						
< = 2	11	7	63.64%	4	36.36%	0.258
< = 5cm	114	52	45.61%	62	54.39%	
> 5cm	17	11	64.71%	6	35.29%	
Invasion into						
chest wall	18	11	61.11%	7	35.29%	
**Lymph node metastasis**						
Negative	93	50	53.76%	43	46.24%	0.35
Positive	67	31	46.27%	36	53.73%	
**Grade**						
I	40	13	32.50%	27	67.50%	0.251
II	39	18	46.15%	21	53.85%	(I vs II + III)
III	1	0	0.00%	1	100.00%	
unknown	80	50	62.50%	30	37.50%	

### Expression of PD-L1 on breast cancer cells is up-regulated by IFN-γ

**Figure 1:**
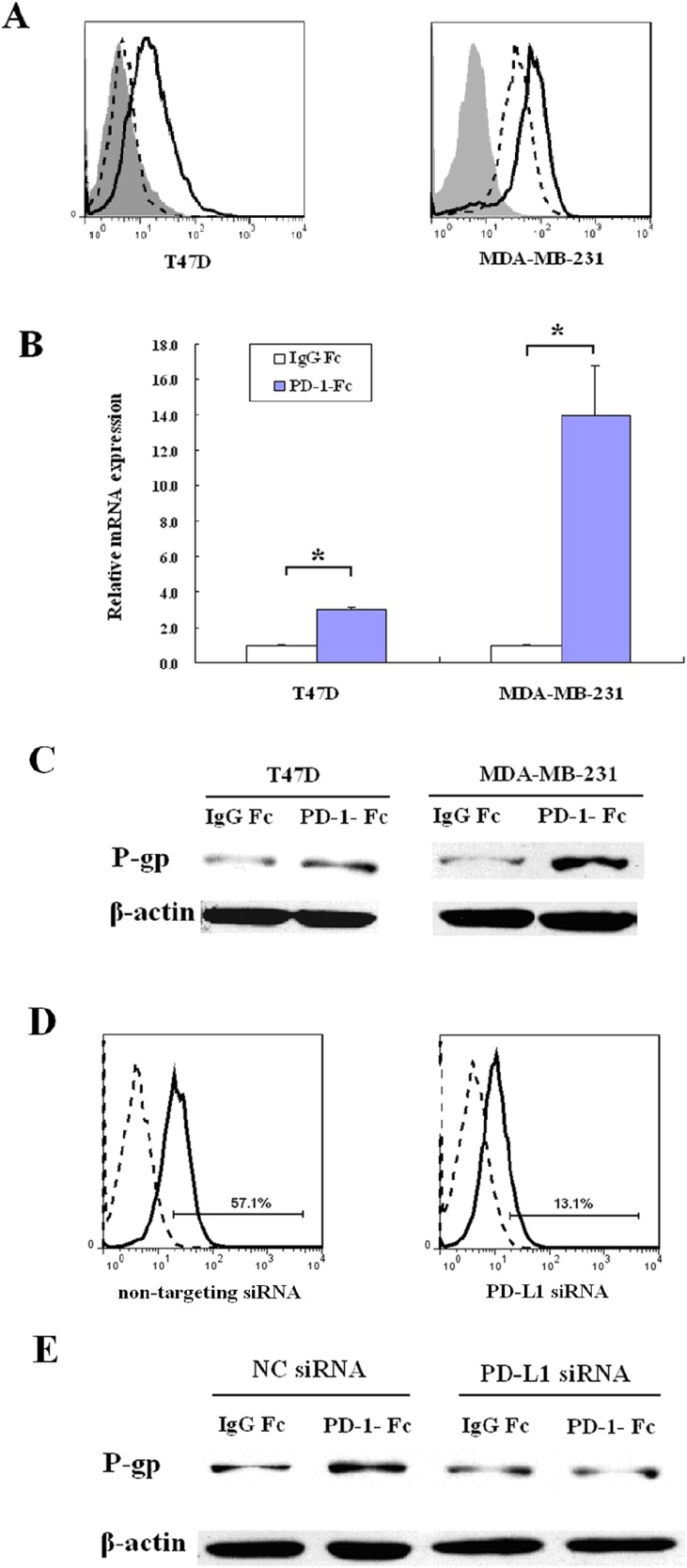
PD-1/PD-L1 interaction up-regulates MDR1/P-gp expression in breast cancer cell lines **(A**) T47D and MDA-MB-231 cells were treated with 75ng/ml IFN-γ for 24 hours and the cell surface expression of PD-L1 was determined by flow cytometry. The shaded histograms indicate staining with PE-labeled isotype control IgG, the dashed histograms indicate staining with PE-labeled anti-PD-L1 antibody, and the open histograms indicate IFN-γ treated cells staining with PE-labeled anti-PD-L1 antibody. (**B**) MDR1 mRNA was analyzed by real-time quantitative PCR in MDA-MB-231 cells or IFN-γ-treated T47D cells after treatment with IgG Fc or PD-1-Fc for 24 h. Amplification of β-actin mRNA was used as a control.(^*^p <0 .05). (**C**) MDR1/P-gp was determined in MDA-MB-231 cells or IFN-γ-treated T47D cells by western blot after treatment with IgG Fc or PD-1-Fc for 24 h. (**D**) Flow cytometric analysis of PD-L1 on MDA-MB-231 after treatment with non-targeting siRNA or PD-L1 siRNA. The dashed histograms indicate staining with PE-labeled isotype control IgG, the solid histograms indicate staining with PE-labeled anti-PD-L1 antibody. (**E**) After knockdown of PD-L1, MDA-MB-231 was treated with PD-1-Fc for 24 h and MDR1/P-gp was determined by western blot. Non-targeting siRNAs were used as negative controls (NC).

Breast cancer cell lines T47D and MDA-MB-231 were analyzed for PD-L1 expression by flow cytometry. T47D cells express undetectable cell surface levels of PD-L1; however, the PD-L1 expression is induced by 24h incubation with IFN-γ. MDA-MB-231 constitutively expresses high levels of PD-L1, which is further increased by 24h treatment with IFN-γ (Figure 1A).

### PD-1/PD-L1 interaction increases MDR1/P-gp expression in breast cancer cells

Since we have shown that PD-L1 expression correlates with MDR1/P-gp expression in breast cancer tissues, we analyzed whether PD-L1 up-regulates MDR1*/*P-gp in breast cancer cells, in the presence of PD-1. Incubation of MDA-MB-231 cells with PD-1-Fc for 24h increased the MDR1/P-gp mRNA level 13.95–fold (*p* < 0.05) compared with IgG1 Fc control. Exposure of IFN-γ-treated (24 h) T47D cells to PD-1-Fc increased the MDR1/P-gp mRNA level 3.03–fold (*p* < 0.05; Figure 1B). The amplified products were confirmed by agarose gel electrophoresis and sequencing (data not shown). Incubation of T47D and MDA-MB-231 cells with PD-1-Fc also increased the MDR1/P-gp protein levels (Figure 1C). We investigated the effect of IFN-γ on MDR/P-gp expression in the present study. The result showed that IFN-γ did not induce MDR/P-gp expression in T47D (data not shown).

Since MDA-MB-231 cells constitutively express PD-L1, they were used to investigate the PD-L1/PD-1 interaction. To confirm that the up-regulation of MDR1/P-gp was due to the interaction of PD-1 and PD-L1, PD-L1 was transiently knocked down using a specific siRNA (Figure 1D). As shown in Figure 1E, PD-1-Fc failed to up-regulate MDR1/P-gp after PD-L1 suppression in MDA-MB-231 cells, indicating that the interaction of PD-L1 and PD-1 increases MDR1/P-gp expression in TNBC cells.

### Up-regulation of MDR1/P-gp is dependent on PI3K/AKT and MAPK/ERK pathways

**Figure 2:**
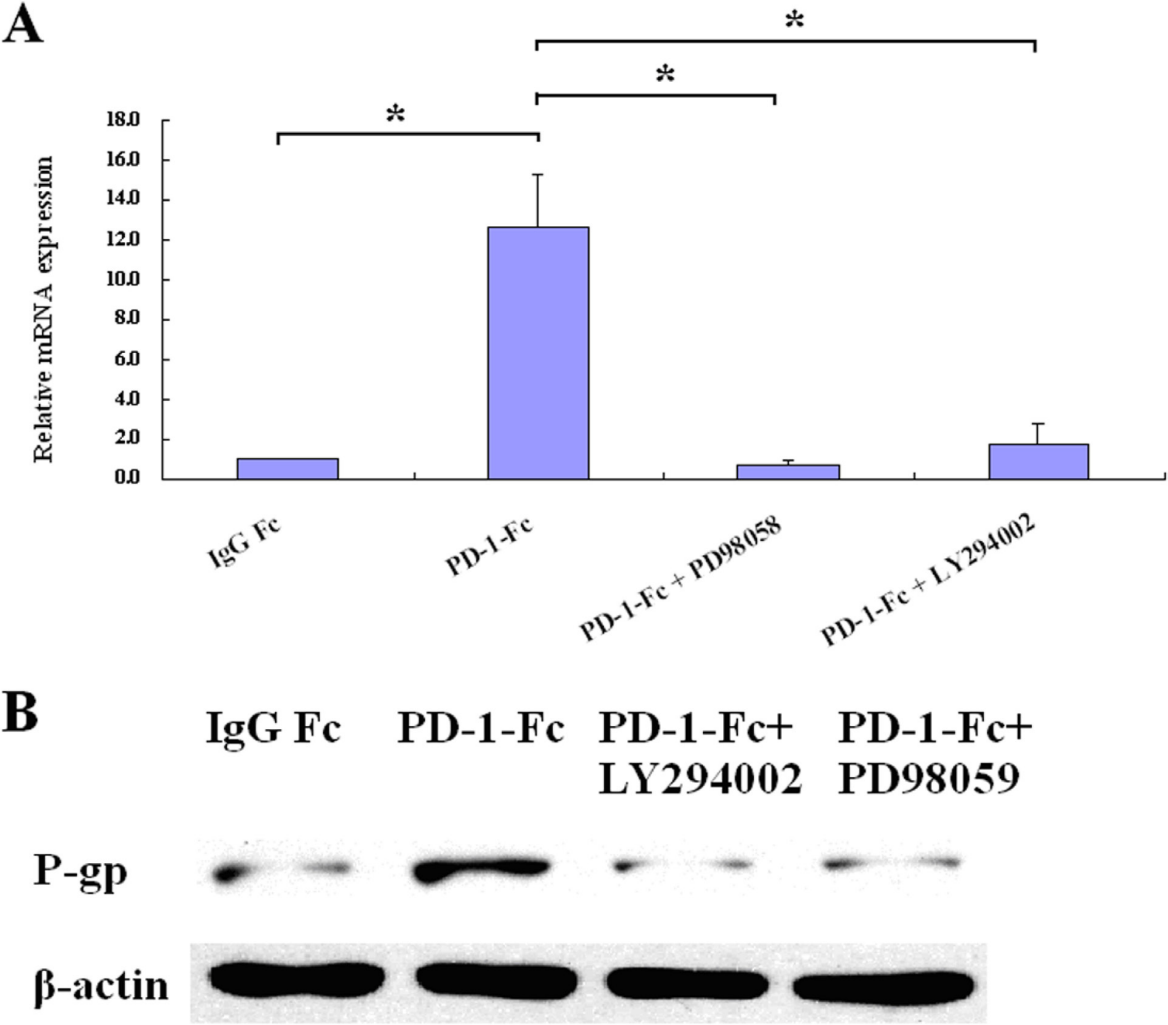
PI3K/AKT and MAPK/ERK inhibitors abrogate PD-L1-mediated MDR1/P-gp up-regulation (**A**) MDR1 mRNA was analyzed by real-time quantitative PCR in MDA-MB-231. Cells were pretreated with each inhibitor for 2 hours prior to treatment of PD-1-Fc (^*^p < 0.05). (**B**) MDR1/P-gp was determined by western blot after treatment with PD-1-Fc for 24 h in present or absent of inhibitors.

As it was reported that PI3K/AKT and MAPK/ERK pathways are involved in the regulation of MDR1/P-gp expression, we investigated their roles in the PD-L1-mediated MDR1/P-gp up-regulation in MDA-MB-231 cells. As shown in Figure 2A, MAPK/ERK inhibitor (PD98059, 10 µM) inhibited the up-regulation of MDR1 mRNA. PI3K/AKT pathway inhibitor (LY294002, 10 µM) had a lesser inhibition effect on the up-regulation of MDR1 mRNA. Similar results were observed on protein levels (Figure 2B). As PI3K/AKT and MAPK/ERK pathways were reported to contribute to PD-L1 expression in breast cancer cells, inhibition of these pathways might have suppressed the PD-L1 expression. To exclude the possibility that the down-regulation of MDR1/P-gp was due to the inhibition of PD-L1 expression by PI3K/AKT and MAPK/ERK pathways, MDA-MB-231 cells were treated with LY294002 (10 µM) or PD98059 (10 µM) and analyzed for PD-L1 expression. As shown in Supplementary Figure 2, LY294002 or PD98059 treatment did not down-regulate the PD-L1 expression in MDA-MB-231 cells, indicating that the up-regulation of MDR1/P-gp is due to the activation of PI3K/AKT and MAPK/ERK pathways.

### PD-1/PD-L1 binding activates PI3K/AKT and MAPK/ERK signaling in MDA-MB-231 cells

**Figure 3:**
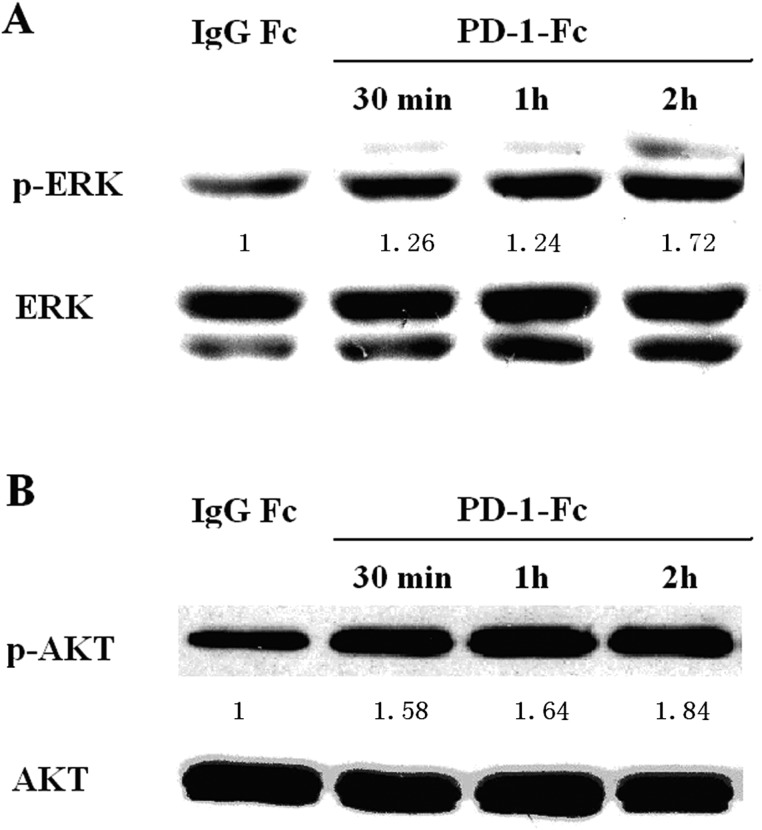
Effect of PD-1/PD-L1 interaction on phosphorylated ERK (p-ERK) and phosphorylated AKT (p-AKT) in MDA-MB-231 (**A**) p-RRK and total ERK were determined by western blot after treatment with IgG Fc or PD-1-Fc for indicated periods. (**B**) p-AKT and total AKT were determined by western blot after treatment with IgG Fc or PD-1-Fc for indicated periods. Numbers represent p-ERK or pAKT relative signal intensities.

To determine whether PD-1/PD-L1 binding activates the PI3K/AKT and MAPK/ERK pathways in MDA-MB-231 cells, MDA-MB-231 cells were treated with PD-1-/Fc, and AKT and ERK activities were analyzed by Western blot analysis. As shown in Figures 3A and 3B, phosphorylated levels of AKT and ERK were increased in MDA-MB-231 cells stimulated with PD-1-/Fc.

### PD-1/PD-L1 interaction increases survival of MDA-MB-231 cells following exposure to doxorubicin

**Figure 4:**
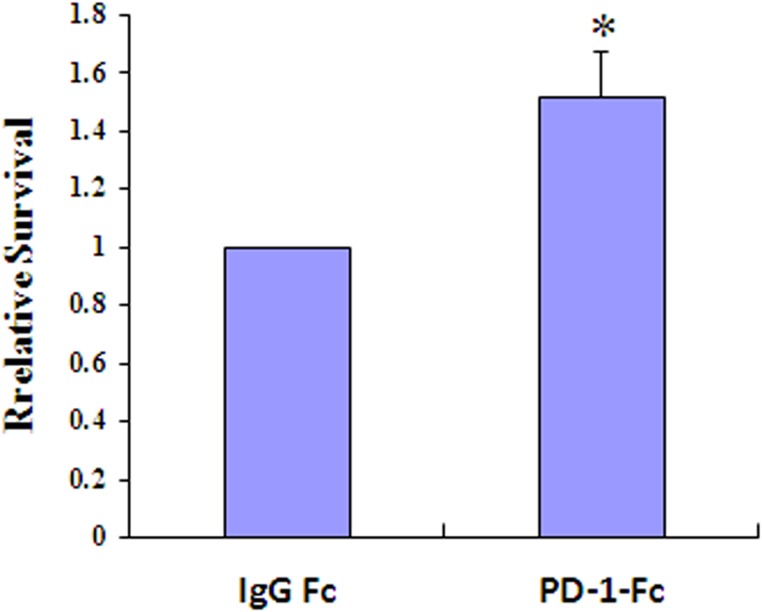
PD-1/PD-L1 interaction results in increased resistance to doxorubicin Results of clonogenic assays using MDA-MB-231 cells incubated with recombinant PD-1-Fc (0.2 µg/ml) for 24 h prior to exposure to doxorubicin (1 µM) (^*^p < 0.05).

To investigate the contribution of PD-1/PD-L1 interaction to drug resistance, MDA-MB-231 cells were incubated with PD-1-Fc for 24 h prior to exposure to doxorubicin, and their survival was analyzed. As shown in Figure 4, PD-1/PD-L1 interaction increased survival of MDA-MB-231 cells incubated with doxorubicin (*p* < 0.05).

## DISCUSSION

In this study, we found that the PD-L1 expression is increased in breast cancer tissues, and correlates with the expression of MDR1/P-gp. The increased expression of PD-L1 also correlates with lymph node metastases in breast cancer patients, histological grade of tumors, and their Her-2 status. Interaction of PD-L1 with PD-1 increases PI3K/AKT and MAPK/ERK signaling in breast cancer cells, resulting in the increased MDR1/P-gp expression.

PD-L1 is up-regulated in many types of tumors. Previous studies mainly focused on the function of PD-L1 as a ligand that interacts with PD-1 on T cells. This interaction results in the inhibitory signal delivered to T cells, resulting in apoptosis and exhaustion of T cells [23–25]. Several reports have indicated that PD-L1 can deliver a reverse signal to cancer cells, and act as an anti-apoptotic molecule. In this study, we observed that phosphorylation of AKT and ERK increased following PD-L1/PD-1 interaction induced by PD-1-Fc treatment; this was consistent with previous reports [19]. The PI3K/AKT and MAPK/ERK signaling pathways play a crucial role in tumorigenesis and tumor progression by promoting cell proliferation and inhibiting apoptosis [26, 27]. Activation of these pathways by the PD-L1/PD-1 interaction may contribute to the resistance of cancer cells to chemotherapeutic agents.

Drug resistance refers to the status of poor responsiveness of tumor cells to chemotherapeutic drugs. The main mechanism of drug resistance is overexpression of the ATP-dependent efflux pump, known as P-gp [28], which is the first known member of the ABC transporter superfamily, and is encoded by the *MDR1* gene. In this study, we found a correlation between MDR1/ P-gp and PD-L1 expression in breast cancer tissues. We further showed that the PD-L1 expression on breast cancer cells increased the MDR1/P-gp expression in the presence of PD-1. In MDA-MB-231 cells, knockdown of PD-L1 with siRNA inhibited the MDR1/P-gp up-regulation after PD-1-Fc treatment, demonstrating that the up-regulation of MDR1 was due to the reverse signal derived from PD-L1 on breast cancer cells. To our knowledge, the present work is the first demonstration that PD-L1 reverse signal can up-regulate the expression of MDR1/P-gp.

Previous studies have indicated that PI3K/AKT and MAPK/ERK pathways are involved in the P-gp biosynthesis and degradation [29, 30]. Our study demonstrated that PI3K/AKT and MAPK/ERK pathways were activated by PD-1 and PD-L1 interaction in breast cancer cells. Thus, we hypothesized that the up-regulation of MDR1/P-gp by PD-L1 is mediated by the activation of PI3K/AKT and MAPK/ERK pathways. Indeed, we found that inhibition of PI3K/AKT and MAPK/ERK pathways suppressed the PD-L1-induced MDR1/P-gp up-regulation, indicating that PD-L1 reverse signal up-regulates the MDR1/P-gp expression via activation of PI3K/AKT and MAPK/ERK pathways. Several reports have shown that PD-1 can be expressed as a soluble form [31, 32]. Thus, soluble PD-1 or PD-1 expressed on tumor infiltrating T cells may regulate the MDR1/P-gp expression on breast cancer cells. In addition, we evaluated the chemoresistance function using colony formation assay and the results showed that PD-1/PD-L1 interaction increased survival in MDA-MB-231 cells exposed to PD-1-Fc prior to doxorubicin. However, the mechanisms of how PD-L1 delivers the intracellular reverse signal, and inhibits apoptosis in cancer cells, need further investigation.

Our results have shown that the PD-L1 expression in breast cancer tissues correlates with lymph node metastasis and HER2 expression, which is consistent with previous reports [33, 34]. However, the association of PD-L1 expression with patients’ age, and ER and PR status was different compared to previously published studies [13, 34]. This inconsistency may be attributable to differences in the IHC staining for PD-L1, or the use of tissue microarray (TMA) to assess the PD-L1 expression. TMA allowed us to investigate multiple specimens simultaneously, but the use of TMAs may not accurately represent PD-L1 protein expression due to the intra-tumor heterogeneity of expression. We also showed that high PD-L1 expression levels were associated with low-grade tumor. The results in this part were only got from the statistical analysis of the IHC experiment. However, the mechanisms involved in this phenomenon are still not completely elucidated in other reports, which need further investigation. In this study we found no significant correlation between MDR/P-g expression and clinicopathological features, which is consistent with previous reports [35–38].But conflicting results have been obtained from several studies [35, 38, 39]. There are also conflicting results regarding the role of MDR1/P-gp on prognosis [36, 37, 40–42] . This may be partly due to different subsets of tumors, the use of different tumor material, and the use of different antibodies. Furthermore, the genetic polymorphism of MDR1 might be associated with prognosis [43, 44].

One of the limitations of this study is that we did not evaluate correlation between PD-L1 and patient prognosis. Other reports have indicated that PD-L1 correlates with poor prognosis in breast cancer patients [3, 11]. Another limitation is that we did not investigate the reproducibility of these data in other PD-L1-positive breast cancer cell lines. Considering the endogenous biological differences in breast cancer cell lines, future studies should include multiple cell lines.

In conclusion, we have shown that PD-L1 binding to PD-1 up-regulates the MDR1/P-gp expression in breast cancer cells, indicating that this may contribute to the resistance to chemotherapy drugs in breast cancer patients. In addition, our results indicate that the PD-L1/PD-1-induced MDR1/P-gp expression is mediated by the activation of PI3K/AKT and MAPK/ERK pathways. Furthermore, we demonstrate that the PD-L1 expression in breast cancer tissues is associated with histological grade of tumors, lymph node metastasis, and HER2 status. Additional studies are needed to elucidate the mechanisms regulating MDR1/P-gp expression in breast cancer cells, and the extent of how MDR1/P-gp contributes to drug resistance. However, our present findings suggest that the PD-1/PD-L1 inhibition may increase chemotherapy efficacy by inhibiting the MDR1/P-gp expression in breast cancer cells.

## MATERIALS AND METHODS

### Cell culture

T47D cells were cultured in RPMI 1640 medium supplemented with 10% FBS, 100 IU/ml Penicillin, and 100 mg/ml Streptomycin at 37°C in 5% CO_2_. MDA-MBA-231 cells were cultured in Dulbecco’s modified Eagle’s medium (DMEM) supplemented with 10% FBS, 100 IU/ ml Penicillin, and 100 mg/ ml Streptomycin.

### Flow cytometry (FACS)

Flow cytometry analysis was used to access the cell surface PD-L1 expression. Cells were harvested and stained with phycoerythrin (PE)-labeled anti-PD-L1 antibody (BD Biosciences) or isotype control IgG for 30 min. The cells were washed with PBS twice and suspended in 500 µl of PBS. The cells were analyzed using BD FACSCaliber flow cytometer (BD Biosciences). FlowJo software (Tree Star Inc.) was used for data analysis.

### Cell treatment with IFN-γ and PD-1-Fc

Cells were seeded in 35 mm wells, incubated 24 h with recombinant human IFN-γ (75 ng/ml, Peprotech) when the monolayer cell density reached ∼70% confluency, and analyzed for PD-L1 expression by flow cytometry. Cells were supplemented with human recombinant PD-1-Fc (1 μg/ml for T47D cells, and 0.5 μg/ml for MDA-MB-231 cells, Genscript) or IgG Fc (Sino Biological) for 24 h and harvested for further analysis.

### Quantitative real-time RT-PCR

Total RNA was extracted using Trizol reagent (Sigma), and RNA concentration was determined using NanoDrop 2000c spectrometer (Thermo Scientific). cDNA was prepared with RevertAid Stand cDNA Synthesis Kit (Thermo Scientific); β-actin was used as an internal control. Levels of human *MDR1* mRNA were assessed using FastStart Universal SYBR Green Master (ROX) (Roche). Real-time PCR was performed on an ABI 7500 Real-Time PCR System. Relative gene expression levels were analyzed using the 2^-ΔΔCt^ method.

### Western blot analysis

Cells were lysed with RIPA lysis buffer (Beyotime Biotechnology). Lysates were cleared by centrifugation at 12,000 g for 5 min at 4 °C and the total protein concentration was determined by BCA Protein Assay Kit (Beyotime Biotechnology). The lysates were separated using SDS-PAGE and transferred onto PVDF membranes. Membranes were blocked in 5% non-fat dry milk in TBST buffer for 1 hour at room temperature. The membranes were incubated with primary antibody followed by HRP-conjugated goat anti-rabbit secondary antibody (ZSGBbio). Bands were visualized using Pro-light HRP Chemiluminescent Kit (Taingen). Primary antibodies against MDR1/P-gp, ERK, p-ERK and p-AKT were purchased from Cell Signaling Technology. β-actin and AKT antibodies were purchased from Bioss.

### Knockdown of PD-L1 by short interfering (si) RNA

PD-L1 expression was knocked down in MDA-MB-231 cells using a specific siRNA (GenePharma). Non-targeting siRNAs were designed as negative controls (NC). Cells were plated in 24-well plates at 5×10^4^ cells per well. After 18 to 24 hours, the monolayer cell density reached to the ∼50% confluency and the PD-L1 siRNA or negative control were transfected using GenMute siRNA Transfection Reagent (Signagen) according to the manufacturer’s instructions. Cells were cultured for 48 h and analyzed for membrane expressions of PD-L1.

### Immunohistochemistry (IHC)

Formalin-fixed and paraffin-embedded (FFPE) tissues from breast cancer patients were analyzed by tissue micro arrays (TMAs). The levels of PD-L1 and MDR1/P-gp in breast cancer samples were assessed using IHC with anti-PD-L1 (Proteintech) and anti-MDR1/P-gp antibodies (Proteintech). Antigen retrieval was performed in pH 6.0 citrate buffer (10 mM trisodium citrate, 0.05% Tween-20) in microwave oven. Slides were incubated with 3% H_2_O_2_ for 30 minutes, followed by serum blocking, and staining with anti-PD-L1 and anti-MDR1/P-gp antibodies overnight at 4°C. Then the slides were incubated with HRP-conjugated secondary anti-rabbit IgG at 37°C for 30 minutes. Slides were visualized with diaminobenzidine (DAB), counterstained with hematoxylin, and mounted. The percentage of stained cells was defined as follows: 0, negative; 1, 1%–10%; 2, 11%–50% and 3, 51%–100% of the cells. Immunostaining intensity was scored as follows: 0, no staining; 1, weak staining; 2, moderate staining; and 3, heavy staining. The immunostaining intensity scores were multiplied by the percent of cells stained to obtain the final staining score. The final staining score less than or equal to 4 was defined as ‘low expression’ and more than 4 was defined as ‘high expression’.

### Clonogenic (colony formation) assay

MDA-MB-231 cells were plated in six-well plates and incubated with PD-1-Fc for 24 h. Then, the cells were incubated with doxorubicin (1 µM) or control DMSO for 1 hr. Cells were then seeded at various densities in triplicates. After 7–14 days, colonies were fixed with methanol, stained with dilute crystal violet, and counted.

### Statistical analysis

Quantification of target changes was performed using the two-tailed Student’s *t* test. Correlation coefficients (r) were calculated using the nonparametric Spearman rank correlation test. Differences with *p* < 0.05 were considered significant. Statistical analyses were performed with SPSS 17.0 statistical software (Chicago, IL, USA).

## SUPPLEMENTARY MATERIALS FIGURES



## References

[1] Ishibashi M, Tamura H, Sunakawa M, Kondo-Onodera A, Okuyama N, Hamada Y, Moriya K, Choi I, Tamada K, Inokuchi K (2016). Myeloma Drug Resistance Induced by Binding of Myeloma B7-H1 (PD-L1) to PD-1. Cancer Immunol Res.

[2] Keir ME, Butte MJ, Freeman GJ, Sharpe AH (2008). PD-1 and its ligands in tolerance and immunity. Annu Rev Immunol.

[3] Qin T, Zeng YD, Qin G, Xu F, Lu JB, Fang WF, Xue C, Zhan JH, Zhang XK, Zheng QF, Peng RJ, Yuan ZY, Zhang L, Wang SS (2015). High PD-L1 expression was associated with poor prognosis in 870 Chinese patients with breast cancer. Oncotarget.

[4] Matsuzaki J, Gnjatic S, Mhawech-Fauceglia P, Beck A, Miller A, Tsuji T, Eppolito C, Qian F, Lele S, Shrikant P, Old LJ, Odunsi K (2010). Tumor-infiltrating NY-ESO-1-specific CD8+ T cells are negatively regulated by LAG-3 and PD-1 in human ovarian cancer. Proc Natl Acad Sci USA.

[5] Inman BA, Sebo TJ, Frigola X, Dong H, Bergstralh EJ, Frank I, Fradet Y, Lacombe L, Kwon ED (2007). PD-L1 (B7-H1) expression by urothelial carcinoma of the bladder and BCG-induced granulomata: associations with localized stage progression. Cancer.

[6] Tamura H, Ishibashi M, Yamashita T, Tanosaki S, Okuyama N, Kondo A, Hyodo H, Shinya E, Takahashi H, Dong H, Tamada K, Chen L, Dan K (2013). Marrow stromal cells induce B7-H1 expression on myeloma cells, generating aggressive characteristics in multiple myeloma. Leukemia.

[7] Riella LV, Paterson AM, Sharpe AH, Chandraker A (2012). Role of the PD-1 Pathway in the Immune Response. Am J Transplant.

[8] Freeman GJ (2008). Structures of PD-1 with its ligands: sideways and dancing cheek to cheek. Proc Natl Acad Sci USA.

[9] Shi L, Chen S, Yang L, Li Y (2013). The role of PD-1 and PD-L1 in T-cell immune suppression in patients with hematological malignancies. J Hematol Oncol.

[10] Chen L (2004). Coinhibitory molecules of the B7-CD28 family in the control of T-cell immunity. Nat Rev Immunol.

[11] Ghebeh H, Mohammed S, Al-Omair A, Qattan A, Lehe C, Al-Qudaihi G, Elkum N, Alshabanah M, Bin Amer S, Tulbah A, Ajarim D, Al-Tweigeri T, Dermime S (2006). The B7-H1 (PD-L1) T lymphocyte-inhibitory molecule is expressed in breast cancer patients with infiltrating ductal carcinoma: correlation with important high-risk prognostic factors. Neoplasia.

[12] Soliman H, Khalil F, Antonia S (2014). PD-L1 Expression Is Increased in a Subset of Basal Type Breast Cancer Cells. Plos One.

[13] Sabatier R, Finetti P, Mamessier E, Adelaide J, Chaffanet M, Ali HR, Viens P, Caldas C, Birnbaum D, Bertucci F (2015). Prognostic and predictive value of PDL1 expression in breast cancer. Oncotarget.

[14] Ghebeh H, Tulbah A, Mohammed S, Elkum N, Bin Amer SM, Al-Tweigeri T, Dermime S (2007). Expression of B7-H1 in breast cancer patients is strongly associated with high proliferative Ki-67-expressing tumor cells. Int J Cancer.

[15] Muenst S, Soysal SD, Gao F, Obermann EC, Oertli D, Gillanders WE (2013). The presence of programmed death 1 (PD-1)-positive tumorinfiltrating lymphocytes is associated with poor prognosis in human breast cancer. Breast Cancer Res Treat.

[16] Mittendorf EA, Philips AV, Meric-Bernstam F, Qiao N, Wu Y, Harrington S, Su X, Wang Y, Gonzalez-Angulo AM, Akcakanat A, Chawla A, Curran M, Hwu P (2014). PD-L1 Expression in Triple Negative Breast Cancer. Cancer Immunol Res.

[17] Ghebeh H, Lehe C, Barhoush E, Al-Romaih K, Tulbah A, Al-Alwan M, Hendrayani SF, Manogaran P, Alaiya A, Al-Tweigeri T, Aboussekhra A, Dermime S (2010). Doxorubicin downregulates cell surface B7-H1 expression and upregulates its nuclear expression in breast cancer cells: role of B7-H1 as an antiapoptotic molecule. Breast Cancer Res.

[18] Azuma T, Yao S, Zhu G, Flies AS, Flies SJ, Chen L (2008). B7-H1 is a ubiquitous antiapoptotic receptor on cancer cells. Blood.

[19] Black M, Barsoum IB, Truesdell P, Cotechini T, Macdonald-Goodfellow SK, Petroff M, Siemens DR, Koti M, Craig AW, Graham CH (2016). Activation of the PD-1/PD-L1 immune checkpoint confers tumor cell chemoresistance associated with increased metastasis. Oncotarget.

[20] Dong L, Lv H, Li W, Song Z, Li L, Zhou S, Qiu L, Qian Z, Liu X, Feng L, Meng B, Fu K, Wang X (2016). Co-expression of PD-L1 and p-AKT is associated with poor prognosis in diffuse large B-cell lymphoma via PD-1/PD-L1 axis activating intracellular AKT/mTOR pathway in tumor cells. Oncotarget.

[21] Dizdarevic S, Peters AM (2011). Imaging of multidrug resistance in cancer. Cancer Imaging.

[22] Kobori T, Harada S, Nakamoto K, Tokuyama S (2014). Mechanisms of P-glycoprotein alteration during anticancer treatment: role in the pharmacokinetic and pharmacological effects of various substrate drugs. J Pharmacol Sci.

[23] Freeman GJ, Long AJ, Iwai Y, Bourque K, Chernova T, Nishimura H, Fitz LJ, Malenkovich N, Okazaki T, Byrne MC, Horton HF, Fouser L, Carter L (2000). Engagement of the PD-1 immunoinhibitory receptor by a novel B7 family member leads to negative regulation of lymphocyte activation. J Exp Med.

[24] Tsushima F, Yao S, Shin T, Flies A, Flies S, Xu H, Tamada K, Pardoll DM, Chen L (2007). Interaction between B7-H1 and PD-1 determines initiation and reversal of T-cell anergy. Blood.

[25] Goldberg MV, Maris CH, Hipkiss EL, Flies AS, Zhen L, Tuder RM, Grosso JF, Harris TJ, Getnet D, Whartenby KA, Brockstedt DG, Dubensky TW, Chen L (2007). Role of PD-1 and its ligand, B7-H1, in early fate decisions of CD8 T cells. Blood.

[26] Fruman DA, Rommel C (2014). PI3K and cancer: lessons, challenges and opportunities. Nat Rev Drug Discov.

[27] Dhillon AS, Hagan S, Rath O, Kolch W (2007). MAP kinase signalling pathways in cancer. Oncogene.

[28] Raaijmakers MH (2007). ATP-binding-cassette transporters in hematopoietic stem cells and their utility as therapeutical targets in acute and chronic myeloid leukemia. Leukemia.

[29] Katayama K, Yoshioka S, Tsukahara S, Mitsuhashi J, Sugimoto Y (2007). Inhibition of the mitogen-activated protein kinase pathway results in the down-regulation of P-glycoprotein. Mol Cancer Ther.

[30] Xie X, Tang B, Zhou J, Gao Q, Zhang P (2013). Inhibition of the PI3K/Akt pathway increases the chemosensitivity of gastric cancer to vincristine. Oncol Rep.

[31] Liu C, Jiang J, Gao L, Wang X, Hu X, Wu M, Wu J, Xu T, Shi Q, Zhang X (2015). Soluble PD-1 aggravates progression of collagen-induced arthritis through Th1 and Th17 pathways. Arthritis Res Ther.

[32] Nielsen C, Ohm-Laursen L, Barington T, Husby S, Lillevang ST (2005). Alternative splice variants of the human PD-1 gene. Cell Immunol.

[33] Ghebeh H, Barhoush E, Tulbah A, Elkum N, Al-Tweigeri T, Dermime S (2008). FOXP3+ Tregs and B7-H1+/PD-1+ T lymphocytes co-infiltrate the tumor tissues of high-risk breast cancer patients: Implication for immunotherapy. BMC Cancer.

[34] Muenst S, Schaerli AR, Gao F, Däster S, Trella E, Droeser RA, Muraro MG, Zajac P, Zanetti R, Gillanders WE, Weber WP, Soysal SD (2014). Expression of programmed death ligand 1 (PD-L1) is associated with poor prognosis in human breast cancer. Breast Cancer Res Treat.

[35] Rybárová S, Hodorová I, Hajduková M, Schmidtová K, Mojzis J, Kajo K, Kviatkovská Z, Plank L, Benický M, Mirossay A, Biros E, Bobrov N, Wagnerová M (2006). Expression of MDR proteins in breast cancer and its correlation with some clinical and pathological parameters. Neoplasma.

[36] Schneider J, Lucas R, Sánchez J, Ruibal A, Tejerina A, Martín M (2000). Modulation of molecular marker expression by induction chemotherapy in locally advanced breast cancer: correlation with the response to therapy and the expression of MDR1 and LRP. Anticancer Res.

[37] Taheri M, Mahjoubi F (2013). MRP1 but not MDR1 is associated with response to neoadjuvant chemotherapy in breast cancer patients. Dis Markers.

[38] Li W, Song M (2014). Expression of multidrug resistance proteins in invasive ductal carcinoma of the breast. Oncol Lett.

[39] Schneider J, Gonzalez-Roces S, Pollán M, Lucas R, Tejerina A, Martin M, Alba A (2001). Expression of LRP and MDR1 in locally advanced breast cancer predicts axillary node invasion at the time of rescue mastectomy after induction chemotherapy. Breast Cancer Res.

[40] Surowiak P, Materna V, Matkowski R, Szczuraszek K, Kornafel J, Wojnar A, Pudelko M, Dietel M, Denkert C, Zabel M, Lage H (2005). Relationship between the expression of cyclooxygenase 2 and MDR1/P-glycoprotein in invasive breast cancers and their prognostic significance. Breast Cancer Res.

[41] Zhu Z, Wang B, Bi J, Zhang C, Guo Y, Chu H, Liang X, Zhong C, Wang J (2013). Cytoplasmic HuR expression correlates with P-gp, HER-2 positivity, and poor outcome in breast cancer. Tumour Biol.

[42] Chekhun VF, Zhylchuk VE, Lukyanova NY, Vorontsova AL, Kudryavets YI (2009). Expression of drug resistance proteins in triple-receptor-negative tumors as the basis of individualized therapy of the breast cancer patients. Exp Oncol.

[43] Kim HJ, Im SA, Keam B, Ham HS, Lee KH, Kim TY, Kim YJ, Oh DY, Kim JH, Han W, Jang IJ, Kim TY, Park IA (2015). ABCB1 polymorphism as prognostic factor in breast cancer patients treated with docetaxel and doxorubicin neoadjuvant chemotherapy. Cancer Sci.

[44] Wang Z, Wang T, Bian J (2013). Association between MDR1 C3435T polymorphism and risk of breast cancer. Gene.

