# Supplementary material for: PD-1/PD-L1 interaction up-regulates MDR1/P-gp expression in breast cancer cells via PI3K/AKT and MAPK/ERK pathways
Source: Oncotarget. 2017 Oct 20;8(59):99901–12. doi: 10.18632/oncotarget.21914 (PMC5725139; doi:10.18632/oncotarget.21914)
Supplement: Supplementary file 1 [file oncotarget-08-99901-s001.pdf]

PD-1/PD-L1 interaction up-regulates MDR1/P-gp expression in breast cancer cells via PI3K/AKT and MAPK/ERK pathways

SUPPLEMENTARY MATERIALS

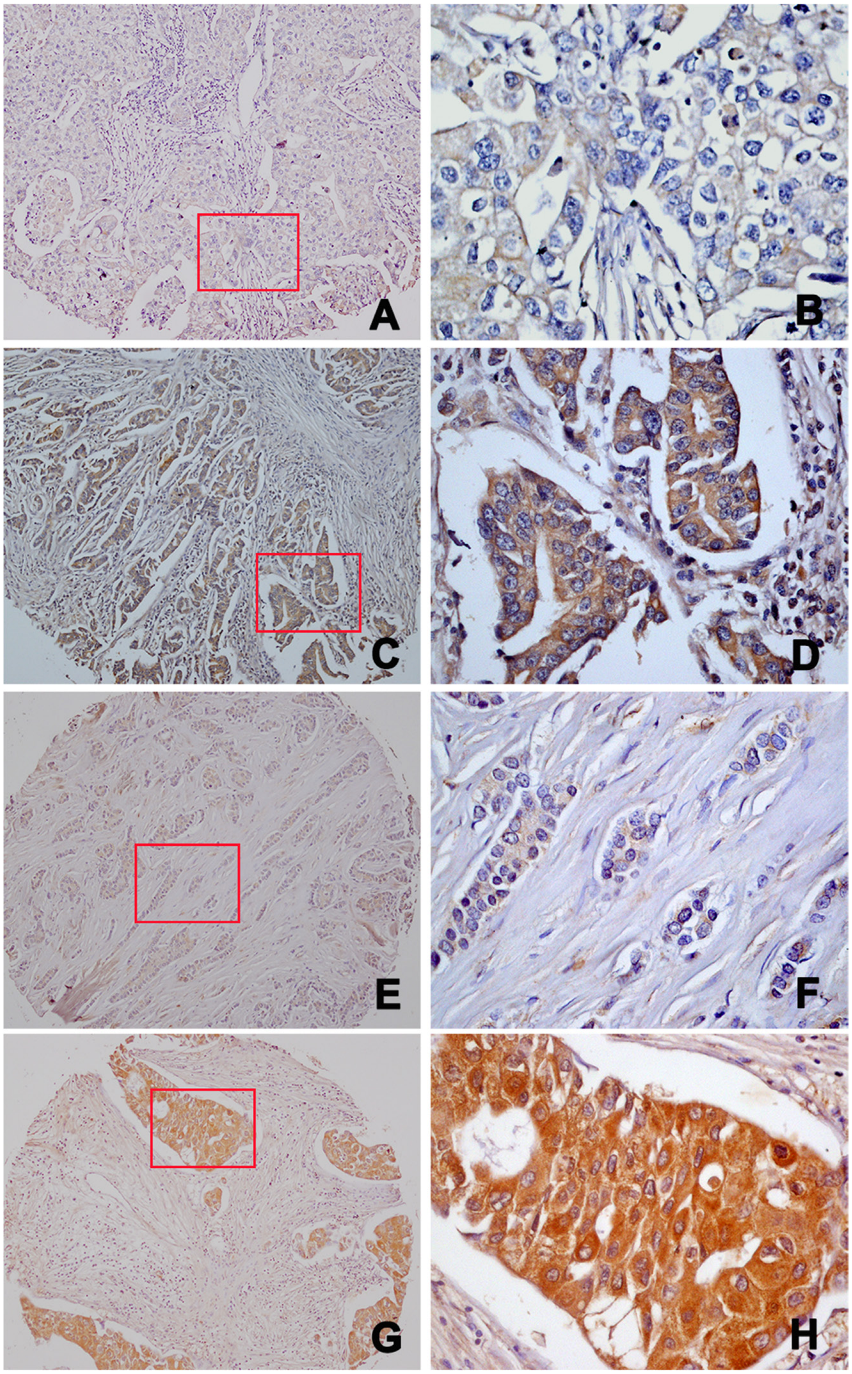

Supplementary Figure 1: Expression of PD-L1 and MDR1/P-gp in breast cancer tissues. (**A**) Low expression of PD-L1 (× 100). (**B**) Low expression of PD-L1 (× 400). (**C**) High expression of PD-L1 (× 100). (**D**) High expression of PD-L1 (× 400). (**E**) Low expression of MDR1/P-gp (× 100). (**F**) Low expression of MDR1/P-gp (× 400). (**G**) High expression of MDR1/P-gp (× 100). (**H**) High expression of MDR1/P-gp (× 400).

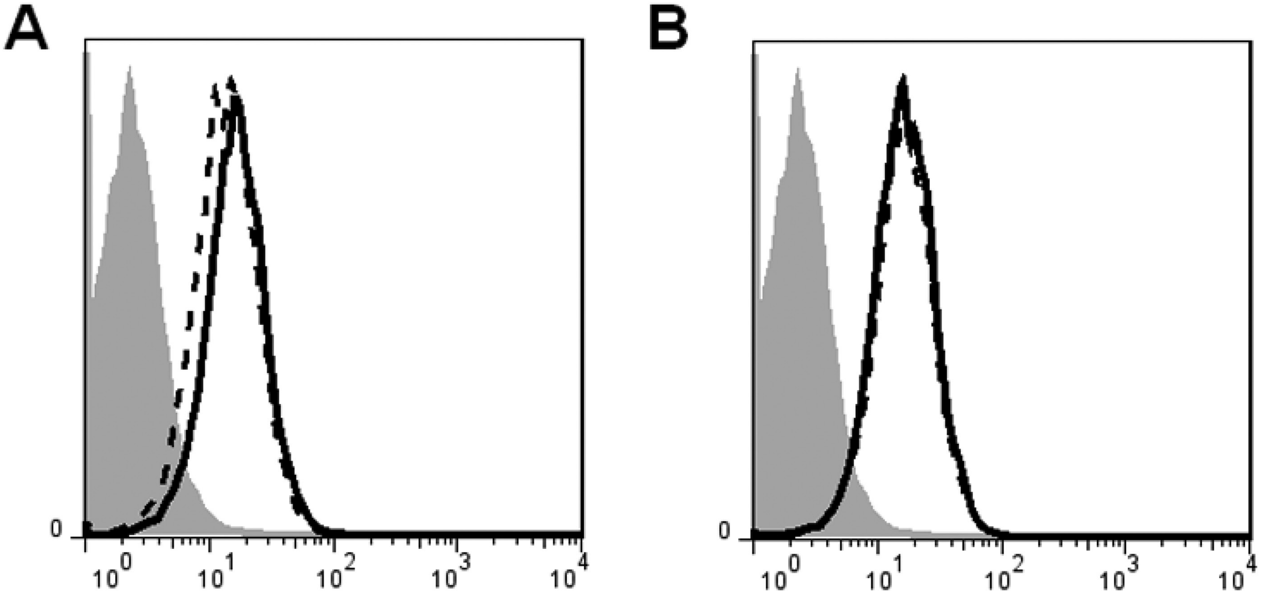

Supplementary Figure 2: PD-L1 expression of MDA-MB-231 after treatment with LY294002 and PD98059. (**A**) PD-L1 expression of MDA-MB-231 after treatment with LY294002. (**B**) PD-L1 expression of MDA-MB-231 after treatment with PD98059. The shaded histograms indicate staining with PE-labeled isotype control IgG, the solid histograms indicate staining with PE-labeled anti-PD-L1 antibody, and the dashed histograms indicate inhibitors treated cells staining with PE-labeled anti-PD-L1 antibody.
